# Recent drug development of dorzagliatin, a new glucokinase activator, with the potential to treat Type 2 diabetes: A review study

**DOI:** 10.1111/1753-0407.13563

**Published:** 2024-05-23

**Authors:** Yu Jiang, Luyao Wang, Zhenhua Dong, Baotian Xia, Shuguang Pang

**Affiliations:** ^1^ School of Clinical Medicine Shandong Second Medical University Weifang China; ^2^ Department of Endocrinology Central Hospital Affiliated to Shandong First Medical University Jinan China

**Keywords:** activating agent of glucokinase, dorzagliatin, Type 2 diabetes

## Abstract

Type 2 diabetes mellitus (T2DM) is a complicated disease related to metabolism that results from resistance to insulin and sustained hyperglycemia. Traditional antidiabetic drugs cannot meet the demand of different diabetes patients for reaching the glycemic targets; thus, the identification of new antidiabetic drugs is urgently needed for the treatment of T2DM to enhance glycemic control and the prognosis of patients suffering from T2DM. Recently, glucokinase (GK) has attracted much attention and is considered to be an effective antidiabetic agent. Glucokinase activators (GKA) represented by dorzagliatin could activate GK and mimic its function that triggers a counter‐regulatory response to blood glucose changes. Dorzagliatin has shown great potential for glycemic control in diabetic patients in a randomized, double‐blind, placebo‐controlled Phase 3 trial (SEED study) and had a favorable safety profile and was well tolerated (DAWN study). In the SEED study, dorzagliatin significantly reduced glycosylated hemoglobin (HbA1c) by 1.07% and postprandial blood glucose by 2.83 mol/L, showing the great potential of this drug to control blood glucose in diabetic patients, with good safety and good tolerance. An extension of the SEED study, the DREAM study, confirmed that dorzagliatin monotherapy significantly improved 24‐h glucose variability and increased time in range (TIR) to 83.7% over 46 weeks. Finally, the clinical study of dorzagliatin combined with metformin (DAWN study) confirmed that dorzagliatin could significantly reduce HbA1c by 1.02% and postprandial blood glucose by 5.45 mol/L. The current review summarizes the development of GK and GKA, as well as the prospects, trends, applications, and shortcomings of these treatments, especially future directions of clinical studies of dorzagliatin.

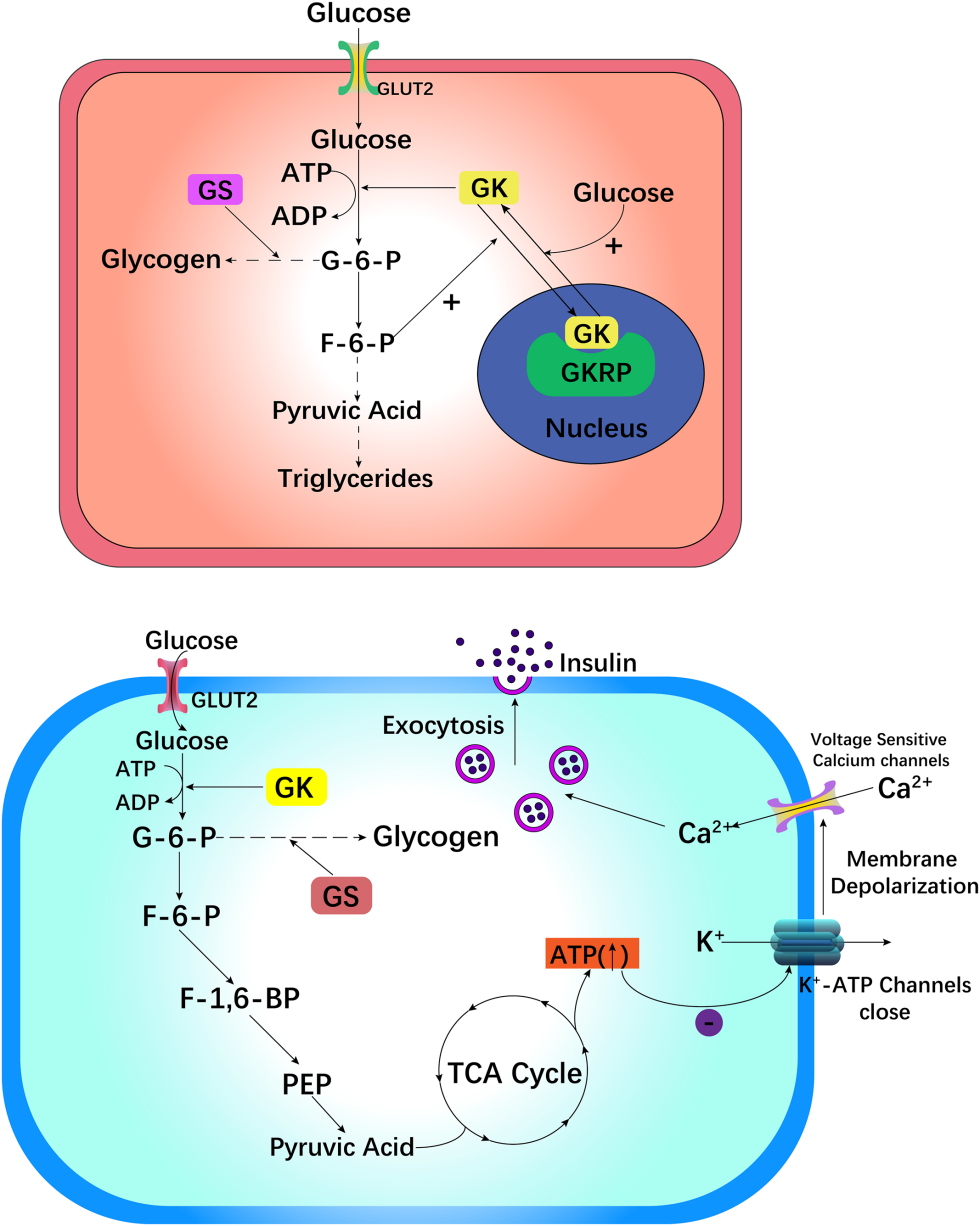

## Graphical abstract

Highlights

- This article is a retrospective review article, aiming to briefly review the working principle of glucokinase (GK) and summarize the development of glucokinase activators (GKA), a new antidiabetes drug targeting GK. In this review, we focus on the development of the new GKA, dorzagliatin, and summarize the results of the latest controlled clinical trials of dorzagliatin. At the same time, we also point out the current limitations of dorzagliatin and the future development direction of dorzagliatin.

## 1 INTRODUCTION

### 1.1 Type 2 diabetes mellitus

#### 1.1.1 Overview of Type 2 diabetes mellitus

Type 2 diabetes mellitus (T2DM), an intricate disease related to metabolism, is caused by complex interactions between genes and circumstances. T2DM is characterized by chronic hyperglycemia accompanied by resistance to insulin and impaired β‐cell function and commonly leads to insulin deficiency. This relative lack of insulin is related to several elements, such as impaired insulin activity, decreased glucose utilization, and aberrant secretion of glucagon; thus, the exact mechanism of T2DM is complex.^1^, ^2^, ^3^, ^4^ Repair of glucose‐stimulated insulin secretion (GSIS) is the key step in the remedy of T2DM. Multiple prospective studies have confirmed that the sensitivity of β cells in the pancreas to glucose was reduced by 50%–70% in T2DM patients, which is the core cause of T2DM.^5^, ^6^

Over 425 million people in the world are affected by diabetes, which represents one of the world's greatest health concerns in the 21st century.^7^, ^8^ A total of 90% of diabetes cases are T2DM, the incidence of which continues to rise.^8^, ^9^ Chronic hyperglycemia is associated with numerous health issues, including cardiovascular disease, neuropathy, and renal and eye disease, which seriously influence quality of life.^7^ Blood glucose control is indispensable in the treatment of diabetes and is associated with a decreased incidence of complications.^10^ The increasing loss of β cells is a main feature during the evolutionary history of this disease. A better understanding of the main disease‐causing pathways would be conducive to the development of drugs that interfere with these processes.^1^, ^11^

#### 1.1.2 Main clinical drugs and their limitations in T2DM

At present, there are nine kinds of hypoglycemic drugs in clinical use, involving insulin (insulin glargine), metformin, glucagon‐like peptide 1 (GLP‐1) receptor agonist (liraglutide), sodium glucose cotransporter 2 (SGLT2) suppressants, dipeptidyl peptidase IV (DPP‐IV) suppressants and other therapeutic drugs.^12^ Although there are a variety of commercially available drugs for T2DM, maintaining optimal control of blood glucose is still difficult. Because of the progressive essence of diabetes, patients generally require a novel hypoglycemic agent every 3–4 years, and glycemia frequently stays at an uncontrollably high level even with combination treatment. Several diabetic patients need several medications and suffer from a variety of adverse effects, including disruption of the digestive system and infection of the genitourinary system.^13^ Moreover, patient treatment options may be limited by positive cardiovascular benefits and few side effects on weight or hypoglycemia. Furthermore, no treatment alters the normal course of β‐cell dysfunction. The most commonly used insulin secretagogue, sulfonylureas, has less glycemic durability than insulin sensitizers according to a study that has cast doubt on the glycemic durability of many routinely used medications.^14^ As a result, efforts are underway to develop drugs that can effectively decrease blood glucose levels, ameliorate the course of the disease, and delay the development of β‐cell failure.

### 1.2 Glucokinase

#### 1.2.1 Summary of glucokinase

Glucokinase (GK) belongs to the hexokinase family and is known as hexokinase IV.^15^, ^16^, ^17^ In general, GK is expressed only in the brain, pancreas, liver, stomach, and intestine, which are all involved in glucose perception^18^, ^19^ Under the action of GK, glucose is phosphorylated on C6 using magnesium adenosine triphosphate (ATP) as a second base to generate glucose‐6‐phosphate (G6P), which is essential for blood glucose regulation.^15^, ^16^, ^20^, ^21^ GK is a glucose sensor in islet alpha cells that regulates glucagon release, and glucokinase activators (GKA) could inhibit glucagon secretion at stimulatory glucose concentrations. In addition, upon inhibition of glucose, GK knockdown leads to a small but meaningful augment in glucagon release, resulting in a complete loss of glucose adjustment to the secretion of glucagon. Therefore, GK plays a crucial role in combining glucose absorption and metabolism with glucagon release.^22^

#### 1.2.2 Function and activation mechanism of GK in the liver

**FIGURE 1:**
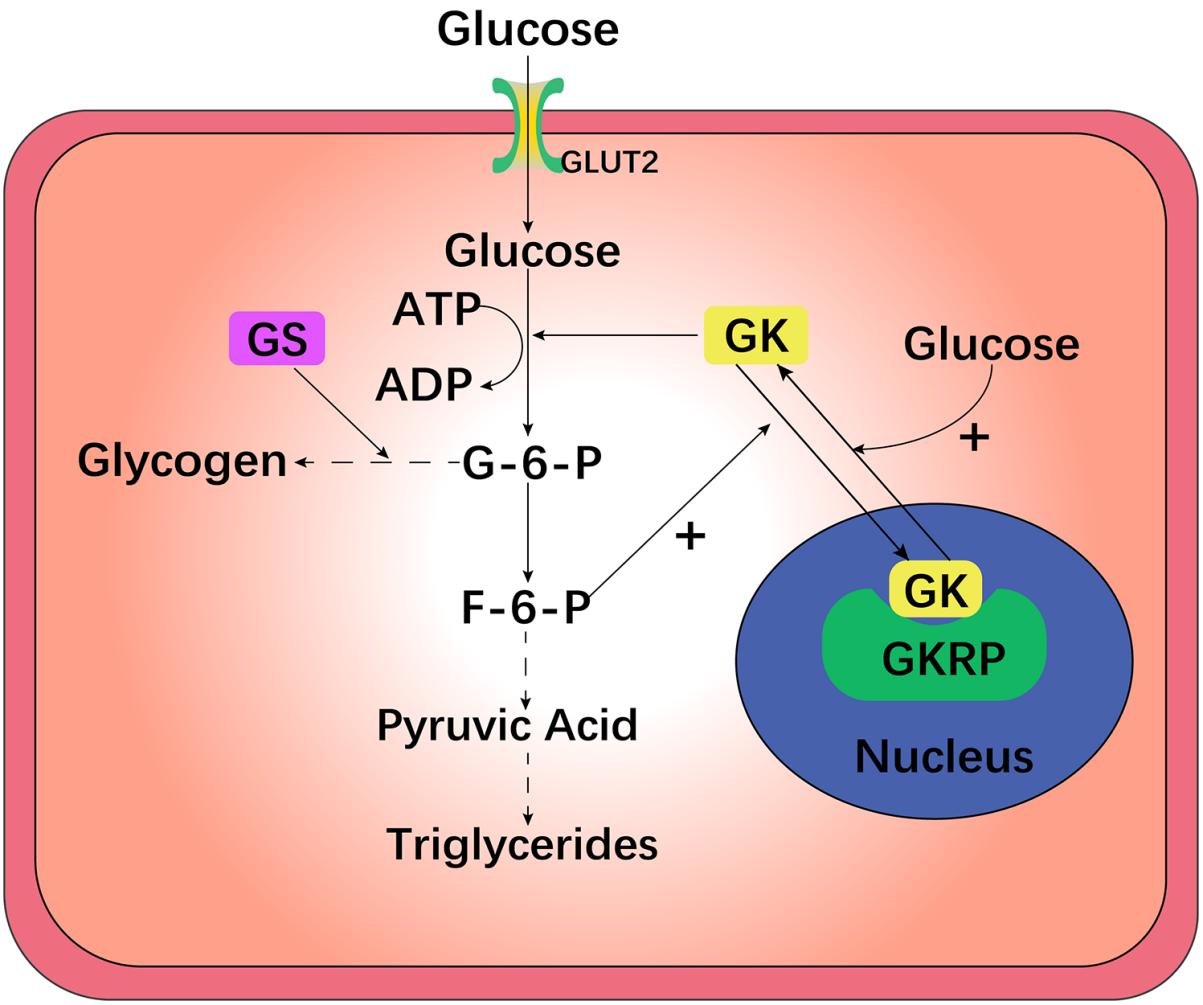
A diagram of the mechanism of GK in hepatic cells. GK, Glucokinase; GKRP, Glucokinase modulatory protein; G‐6‐P, glucose‐6phosphate; F‐6‐P, fructose‐6‐phosphate; F‐1 and 6‐BP, fructose‐1,6‐diphosphate; PEP, phosphoenolpyruvate; GLUT2, glucose translocator 2; GLUT2, glycogen synthase.

GK in liver cells primarily controls glycogen. Insulin and glucagon alter the GK concentration in the cytoplasm of liver cells and regulate the glucose concentration in the cell. Thus, the rate of glucose conversion directly depends on GK activity, as shown in Figure 1.^23^, ^24^

The activity of GK in the liver is adjusted by glucokinase regulatory protein (GKRP). At low glucose concentrations, GK is oriented in the cell nucleus, where it forms an inert GK–GKRP composite with GKRP. When the blood glucose concentration increases, a large amount of glucose enters the cell via glucose translocator 2 (GLUT2), destroys the structure of the GK–GKRP composite, activates the liveness of GK, and enables GK to enter the cytoplasm to function.^25^, ^26^, ^27^, ^28^

Then, liver GK phosphorylates glucose to G6P, which includes absorbing glucose, storing it as glycogen, and inhibiting the synthesis of liver glucose (gluconeogenesis), resulting in a lower concentration of blood sugar.^29^ During the process of phosphorylating glucose to G6P, glcolysis and oxidative phosphorylation occur, followed by the release of insulin. Hence, the rate of glucose phosphorylation to G6P via GK limits the rate of glucose absorption in β cells of the pancreas and becomes the rate‐determining step in the release of insulin. Compared with that of hexokinase, the phosphorylated product G6P has a lesser effect on the reactivity of GK due to its lower molecular weight and higher Michaelis–Menten constant (km) value, which ensures the complete phosphorylation of blood sugar in the physiological range.^23^

#### 1.2.3 Function and activation mechanism of GK in the pancreas

**FIGURE 2:**
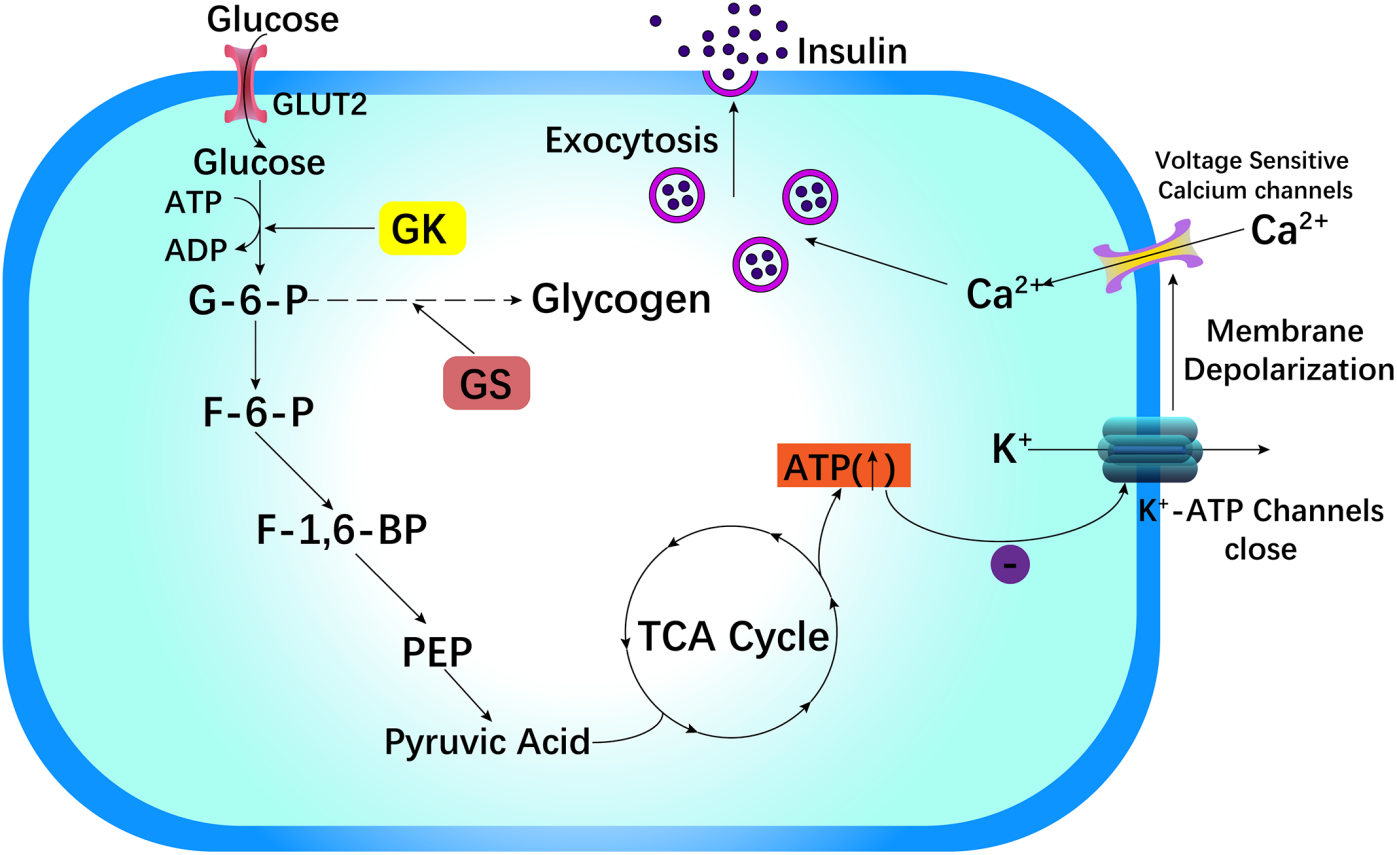
Mechanism of action of GK in pancreatic cells. GK, Glucokinase enzyme; G‐6‐P, glucose‐6‐phosphate; F‐6‐P, fructose‐6‐phosphate; F‐1,6‐BP, fructose 1,6 bisphosphate; PEP, phosphoenolpyruvate; GLUT2, glucose transporter 2; and the TCA cycle, tricarboxylic acid cycle.

As the “glucose receptor” in pancreatic β cells, GK can regulate insulin release according to glucose levels.^30^, ^31^ In pancreatic β cells, GLUT2 mediates the transmembrane transfer of glucose. The quantity and rate of glucose transport are restricted by the rate at which glucose is converted to G6P via the GK enzyme. The process of phosphorylating glucose to G6P is involved in the production of glycogen, which is converted to pyruvate in β cells of the pancreas via a sequence of oxidation–reduction reactions. The involvement of pyruvate in the tricarboxylic acid (TCA) cycle and a rise in electron transport leads to an increase in the ratio of ATP to adenosine diphosphate (ADP), which blocks K^+^‐ATP channels, causes membrane depolarization, and promotes calcium channel sensitivity to voltage opening, leading to a dramatic increase in intracellular Ca^2+^ levels. Finally, Ca^2+^ activates vesicles containing insulin particles, causing insulin to be released into the blood circulation via exocytosis (Figure 2).^6^, ^18^, ^25^, ^26^, ^28^, ^32^, ^33^

A high‐fat diet‐induced diabetic mouse model suggested that improving GK expression in β cells can restore GSIS, decrease blood glucose under fasting conditions, and improve tolerance to glucose, which demonstrated a key effect on impacting the expression of β cell GK in diabetes induced by diet.^33^

### 1.3 Diabetes and GK

Blood glucose receptors in healthy (physiological) conditions respond to variations in blood glucose concentration and coordinate the activity of apparatuses and hormones to normalize blood glucose content.^23^

There is an inverse correlation between the blood glucose level and GK in the liver, and hyperglycemia develops at low GK levels. High‐level GK expression also causes hypoglycemia in the liver for a short period, but it is also related to an incremental risk of glucose intolerance over the long term.^19^, ^23^, ^34^ Knockdown of GK using a precise expression sequence in islet cells produced severe diabetes in mice and led to death in infancy. Thus, GK in islet cells is essential for maintaining glucose homeostasis.^35^

## 2 GLUCOKINASE ACTIVATOR

### 2.1 Development of GKA

GK was identified in liver cells in 1963 and in pancreatic β cells in 1968. It took nearly three decades to realize that GK can act as a drug target for the exploitation of oral antidiabetic agents. Initially, the crucial effect of GK on glucose homeostasis was not accepted, and some studies could not imagine how agonists could activate GK similarly well in β cells in the pancreas and cells in the liver to maximize the effects of reducing glucose without any severe side effects, such as hyperlipidemia. However, the correlation between the expression and stability of GK in pancreatic β cells and T2DM is unclear, and the findings of these studies suggest that target molecules are involved in or decrease in the development of this disease. Given this possibility, the decision to focus on GK in the early 1990s was courageous, given the enormous risks involved in choosing any novel drug objective for research and development. The kinetics and physiological chemistry of GK are well established, and our understanding of its genetics, biochemistry, and biophysics has evolved significantly to establish GK as a novel pharmacological target.^36^, ^37^

The major developments of GKA are summarized in Table 1.

**TABLE 1:** Development of a major glucokinase activator (GKA).

Compound (GKA)	Reference	Duration (weeks)	Dose (mg)	Concomitant drugs	Clinical status	Control	Primary effects	Major adverse events
Piragliatin	38	8 days	25 100	None	Phase I	Placebo‐controlled	Acute reduction of FPG PPG	None
	39	1	10–100^a^	None	Phase II (discontinued)	Placebo‐controlled	Acute reduction of FPG PPG	Hypoglycemia, hepatotoxicity
MK‐0941^b^	40	30	10–40 tid	Insulin glargine ± metformin	Phase III (discontinued)	Placebo‐controlled	HbA1c drop of 0.5 to −0.8% (not sustained), PPG	Hypoglycemia, hypertriglyceridemia, hypertension, loss of efficacy
AZD1656	41	16 + 4	20–200 bid	Metformin	Phase II	Being contrasted with placebo or titrating 5–20 mg glipizide	HbA1c decline of 0.5 to −0.8% (interrupted), PPG	Increase in triglycerides
	42	16	100 tid^a^	None	Phase II	Placebo‐controlled	Nonsignificant HbA1c decline of ~0.2%, FPG	Hypoglycemia, loss of efficacy
PF‐04937319	43	12	100 qd^a^	Add‐on metformin +/− glimepiride	Phase II	Placebo‐controlled or glimepiride	HbA1c decrease of ~0.5%, FPG	Hypoglycemia
	43	12	100 qd^a^	Add‐on metformin +/− glimepiride	Phase II	Placebo‐controlled or glimepiride	HbA1c decline of ~0.7%, FPG	Hypoglycemia
	44	2	150 + 100	None	Phase II	Placebo‐controlled	FPG	None
AMG 151 (ARRY‐403)	45	4	200 bid^a^	Add‐on metformin	Phase II (discontinued)	Placebo‐controlled	FPG	Hypoglycemia, hypertriglyceridemia
TTP399	46	24	400 or 800	Add‐on metformin/sitagliptin	Phase II	Placebo‐controlled	HbA1c drop of ~0.9%	None
GKM‐001	47	2	25–1000	None	Phase II	Placebo‐controlled	FPG	None

Abbreviations: bid, twice a day; FPG, fasting plasma glucose; HbA1c, glycosylated hemoglobin; PPG, postprandial plasma glucose; qd, once a day; tid, three times a day.

^a^ Multiple doses tested.

^b^ To evaluate the efficancy and safety of MK‐0941 in patients with T2DM treated with insulin glargine.

### 2.2 Research status and related clinical trials of dorzagliatin

In 2018, a novel GKA called dorzagliatin, which is an oral and bioavailable GKA that activates GK in the pancreas and liver in a glucose‐dependent manner, was reported to have promising benefits.^48^, ^49^ Dorzagliatin can control hepatic glucose metabolism by regulating the hormone secretion induced by glucose, including pancreatic insulin and GLP‐1 in the enteric canal, and optimizing the signals of hepatic glucose and insulin.^48^, ^49^, ^50^, ^51^ A Phase 2 study involving 258 Chinese participants suffering from T2DM allocated patients to receive an alpha‐glucosidase inhibitor, metformin, or placebo at random. These participants were divided into a placebo group and dorzagliatin groups of 75 mg once a day, 100 mg once a day, 50 mg twice a day, and 75 mg twice a day. Table 2 shows the results of this randomized, double‐blind Phase II clinical test controlled by a placebo. The groups treated with 50 mg twice a day or 75 mg twice a day considerably outperformed the group treated with the placebo at the primary endpoint, as indicated by a decrease in glycosylated hemoglobin (HbA1c) from the beginning to the 12th week. The increase in blood glucose was greater at 2 h after a meal than in the fasting state. Notably, no serious hypoglycemia or harmful side effects were noted. Research and related clinical trials of dorzagliatin demonstrated that dorzagliatin therapy significantly reduced blood glucose and improved the functional parameters of β cells.^23^, ^29^, ^50^

**TABLE 2:** Results of randomized, double‐blind Phase II clinical test controlled by placebo for dorzagliatin.

	Placebo	75 mg qd	100 mg qd	50 mg bid	75 mg bid
Number	53	53	50	50	49
Duration (weeks)	12	12	12	12	12
HbA1c (%)^a^	Drop of 0.35%	Drop of 0.39%	Drop of 0.65%	Drop of 0.79%	Drop of 1.12%
FPG (mmol/L)^a^	Drop of 0.801	Drop of 0.307	Drop of 0.382	Drop of 0.758	Drop of 1.319
2hPPG (mmol/L)^a^	Drop of 1.987	Drop of 4.669	Drop of 4.945	Drop of 3.898	Drop of 4.899
Adverse events	None	3 patients hypoglycemia	2 patients hypoglycemia	3 patients hypoglycemia，1 patient eyelid edema^b^	3 patients hypoglycemia
Severe hypoglycemia	None	None	None	None	None
HOMA‐IR	None	None	None	None	Significant improvement
Disposition index	None	None	None	None	Significant improvement

Abbreviations: 2hPPG, 2‐hour postprandial plasma glucose; bid, twice a day; FPG, fasting plasma glucose; HbA1c, glycosylated hemoglobin; HOMA‐IR, homeostatic model assessment of insulin resistance; qd, once a day.

^a^ Compared with baseline.

^b^ Patients quit the study because of drug‐related harmful events.

In 2020, Zhu et al. conducted the SEED study, which is a randomized, double‐blind, placebo‐controlled Phase III registration study of dorzagliatin monotherapy in 463 patients with newly diagnosed, medication‐naive T2DM. The efficacy and safety of this medicine were studied in patients treated with 75 mg of dorzagliatin twice daily. The first 24‐week, randomized, double‐blind, placebo‐controlled treatment period was used to assess the primary efficacy and safety endpoints of the trial, and the second 28‐week, open‐label treatment period was used to continuously observe and assess the safety of dorzagliatin.^29^, ^52^ A few of the main conclusions of the SEED study include the following:

Significant and long‐lasting effects:

1. HbA1c decreased by 1.07% from baseline to the 24th week, which was obviously better than that in the placebo group.
2. The percentage of patients who achieved the HbA1c target at the 24th week was 42.5%, which was obviously greater than that in the placebo group.
3. Compared with that of the placebo group, β‐cell function was significantly improved, as indicated by a 3.28 increase in homoeostatic model assessment of β‐cell function in T2DM (HOMA2‐β) at Week 24.
4. At the 24th week, the 2‐hour postprandial plasma glucose (2hPPG) concentration decreased by 2.33 mmol/L compared with that in the placebo group.
5. Compared with that of the placebo group, the FPG level decreased by 0.33 mmol/L at 24 weeks.
6. The HbA1c level remained stable at 52 + 6 weeks.

Satisfactory safety and tolerability:

1. The probability of occurrence of hypoglycemia (<3 mmol/L) was less than 1% within 24 weeks, and there were no serious hypoglycemic events.
2. The probability of occurrence of hypoglycemia (<3 mmol/L) was less than 1% within 52 weeks, and there were no serious hypoglycemic events.
3. No severe undesirable events related to the drugs happened within 52 weeks.

Metformin, a primary therapy for T2DM, can reduce fasting plasma glucose levels and hepatic glucose synthesis. After a few years of metformin treatment, other antidiabetic agents often need to be added, and several agents with various mechanisms have been developed as possible additional treatments. Nevertheless, the continual decrease in the function of β cells in T2DM patients is still a challenging issue that needs to be addressed since the conditions of some patients deteriorate to insulin‐dependent diabetes with additional difficulties.^53^, ^54^, ^55^, ^56^

In 2020, Yang, Zhu et al. simultaneously conducted another randomized, double‐blind, placebo‐controlled Phase III registration clinical study, the DAWN study, to investigate the safety and efficacy of dorzagliatin combined with metformin in patients with T2DM who had failed adequate metformin treatment. A total of 767 patients with T2DM were randomly assigned to one of two treatment groups at a 1∶1 ratio: 382 patients in the doxagliptin 75 mg twice daily + metformin 1500 mg·d‐1 group and 385 patients in the placebo twice daily + metformin 1500 mg·d‐1 group. The trial lasted for 52 weeks, with the first 24 weeks of a randomized, double‐blind, placebo‐controlled treatment period for the assessment of the primary efficacy and safety endpoints. The remaining 28 weeks were considered an open treatment period for continuous observation and evaluation of the safety of dorzagliatin. Finally, a total of 751 patients completed this clinical trial. The outcomes of the randomized clinical tests are summarized in Table 3.^48^, ^49^, ^57^

**TABLE 3:** Results of randomized, double‐blind Phase 3 test controlled by placebo plus dorzagliatin in combination with metformin in patients suffering from Type 2 diabetes mellitus (T2DM).

Subject and result	Dorzagliatin + metformin	Placebo + metformin
*n*	376	375
HbA1c (%)		
Baseline	8.3	8.3
24th week	Drop of 1.02	Drop of 0.36
FPG (mmol/L)		
Baseline	9.77	9.95
24th week	Drop of 0.67	Drop of 0.29
2hPPG (mmol/L)		
Baseline	18.82	19.07
Week 24	Drop of 5.45	Drop of 2.97
HOMA2‐β		
Baseline	33.11	31.43
Week 24	3.82	1.40
HOMA2‐IR		
Baseline	1.39	1.49
Week 24	Drop of 0.17	Drop of 0.09

Abbreviations: FPG, fasting plasma glucose; HbA1c, glycosylated hemoglobin; HOMA2‐β, homoeostatic model assessment of β‐cell function in T2DM; HOMA2‐IR, homeostatic model assessment of insulin resistance in T2DM; 2hPPG, 2‐hour postprandial plasma glucose.

The findings of this study demonstrated that adding dorzagliatin can significantly decrease blood glucose levels in T2DM patients, whose conditions cannot be properly managed with metformin alone, while still having a favorable safety and tolerability profile. The following are the main findings of the DAWN study:

Significant and long‐lasting effects:

1. HbA1c decreased by 1.02% from baseline to the 24th week, which was obviously better than that in the group receiving the placebo.
2. The percentage of patients realizing the aim of HbA1c at 24 + 6 weeks was 44.4%, which was obviously greater than that in the placebo group (10.7%).
3. β‐cell function was significantly improved, as indicated by a 2.43 increase in HOMA2‐β at Week 24 in the treated group compared with the placebo group.
4. At the 24th week, the 2hPPG concentration decreased by 2.48 mmol/L compared with that in the placebo group.
5. Compared with that in the placebo group, the FPG in the intervention group decreased by 0.38 mmol/L at 24 weeks.
6. The HbA1c level remained stable at 52 + 6 weeks.

Satisfactory safety and tolerability:

1. The probability of occurrence of hypoglycemia (<3 mmol/L) was less than 1% within 24 weeks, and there were no serious hypoglycemic events.
2. The probability of occurrence of hypoglycemia (<3 mmol/L) was less than 1% within 52 weeks, and there were no serious hypoglycemic events.
3. No severe undesirable events related to the drugs happened within 52 weeks.

In 2023, the DREAM study, a nonpharmacologic observational clinical study initiated by part of the SEED study investigators, was published, which enrolled 69 patients who completed 52 weeks of dorzagliatin treatment and achieved investigator‐assessed glycemia targets (based on investigator‐developed personalized glycemia targets: HbA1c < 7.0% or 7.0% ≤ HbA1c ≤ 8.0%). The primary study was to assess the probability of diabetes remission without taking any hypoglycemia drugs after withdrawal of dorzagliatin. The results showed that the probability of diabetes remission according to the protocol definition reached 65.2% at 52 weeks. In addition, continuous glucose monitoring was used to monitor blood glucose during and after treatment in 16 subjects. Results showed that dorzagliatin treatment significantly improved 24‐h glucose variability and increased time in range (TIR) to 83.7% during 46 weeks of monotherapy in the SEED study phase.^58^

## 3 LIMITATIONS OF THE DORZAGLIATIN STUDY

First, dorzagliatin was designed and registered in China and is available in only one country, which is the greatest limitation. In addition, the latest study evaluated the effectiveness and safety of dorzagliatin as a tailpiece to metformin in patients who are not able to achieve the best control of glycemia with a metformin dose of 1500 mg per day. Another Phase 3 test of the DAWN study^49^ was performed in patients suffering from medium T2DM with glycemic deficiency controlled by metformin only. Hence, these findings may not be applicable to patients suffering from serious T2DM, and further clinical studies are necessary.^48^, ^49^

Second, the current study on the use of dorzagliatin as a supplement to standard treatment with metformin lacks a comparison of the efficacy of dorzagliatin combined with other antidiabetic medicines. The effectiveness and safety of dorzagliatin in combination with various antidiabetic agents must be assessed in international multicenter clinical tests in the future. According to the findings of a recent clinical pharmacological evaluation, dorzagliatin was found to be advantageous for American T2DM patients as a complement to sitagliptin and empagliflozin.^59^ Combination treatment led to a considerable decrease in blood glucose and an increase in GSIS.^59^ Thus, additional clinical studies worldwide are needed to accurately assess the clinical efficacy of dorzagliatin in different T2DM patients and in Chinese populations.

Third, the endurance of placebo observation in a diabetes monotherapy test was restricted to less than 24 weeks due to ethical requirements. Patients in the placebo group were switched to dorzagliatin during the 28‐week period of open‐label therapy. The Phase 3 test of dorzagliatin did not include a control group for the entire 52‐week period because the research was designed to investigate its effectiveness at 24 weeks and safety beyond 52 weeks, and it did not include a control group for open‐label therapy from 24 weeks to 52 weeks. Therefore, an overall period of investigation of 1 year was inadequate to assess the safety and efficacy of dorzagliatin.^48^, ^49^

Fourth, the most recent research data showed no discernible difference between dorzagliatin and placebo in terms of systolic or diastolic blood pressure. The outcome suggested that there is no risk of elevated blood pressure associated with dorzagliatin.^60^ Dorzagliatin does increase triglyceride (TG) and total cholesterol (TC) levels compared with those of the placebo according to meta‐analyses of blood lipids. Trial sequential analysis (TSA) also revealed discrepancies in TG and TC levels. Notably, dorzagliatin led to slight elevations in TG and TC, but these increases were much lower than those caused by earlier GKA^40^, ^60^ and did not increase the risk of hyperlipidemia. Additionally, dorzagliatin did not reduce high‐density lipoprotein cholesterol and increase low‐density lipoprotein cholesterol, which is one of the major elements resulting in an enhancement in significant cardiovascular adverse events.^60^, ^61^, ^62^ However, the potential risk of an increase in TG and TC is a drawback because elevated TG are typical of T2DM and may increase cardiovascular disease incidence. Thus, clinicians should be aware of any possible risks associated with the treatment of elevated TG with dorzagliatin. Future research on the cardiovascular dangers of dorzagliatin is eagerly anticipated.

Finally, asymptomatic hypoglycemic events may have been overlooked.

## 4 CONCLUSION AND PROSPECTS

T2DM is the eighth most common cause of death and disability globally, according to the 2019 Global Burden of Diseases, Injuries, and Risk Factors Study (GBD).^63^, ^64^, ^65^ The International Diabetes Federation (IDF) estimates that there will be 537 million T2DM patients worldwide in 2021.^8^, ^64^, ^65^ T2DM patients have a greater chance of malfunctioning and failing different organs than healthy persons, particularly the kidneys, eyes, and nerves,^63^, ^66^ which leads to a rise in personal medical costs and a loss in quality of life, as well as a significant load on the health‐care system.^65^, ^67^, ^68^, ^69^, ^70^ Diabetes‐related annual expenses are increasing.^8^, ^63^, ^65^ T2DM is also a major risk factor for heart disease and stroke,^63^, ^64^, ^65^ which are the leading causes of death worldwide.^63^, ^65^, ^71^ A report by the World Health Organization predicts that the number of people with diabetes will reach 700 million by 2045.^72^, ^73^ Long‐term medication generally treats diabetes mellitus. Despite the usability of many medications for T2DM therapy, including metformin (a first‐line antidiabetic drug) and traditional oral hypoglycemic agents (sulfonylureas, acarbose, and pyrazolines),^74^, ^75^ many patients have difficulty realizing the best control of this disease.^54^, ^76^, ^77^ In addition, traditional hypoglycemic drugs have difficulty playing an efficient clinical role in treatment due to their poor efficacy and severe side effects.^78^ Therefore, new antidiabetic drugs are urgently needed.

The mechanism of dorzagliatin is to repair the first stage of insulin secretion and improve glucose‐stimulated secretion of GLP‐1 in patients suffering from diabetes through allosteric regulation of GK activity,^79^, ^80^ which can accelerate the synthesis of glycogen in the liver and reduce the output of liver glucose in diabetic patients. This a new mechanism of regulating blood glucose different from traditional hypoglycemic drugs. Dorzagliatin has an amino acid‐like chemical structure and a good linear relationship in human pharmacokinetics. It has no major metabolites and no obvious accumulation in the body. This new structure allows most of the drug to be taken up by the kidney and can be utilized in patients with kidney disease without adjusting the dosage, which provides a new therapeutic option for a large proportion of patients with T2DM, especially patients with renal insufficiency.

In addition, the therapeutic effect of dorzagliatin on diabetes also benefits from new research and development technology, which can effectively improve the exposure of target organs and exert synergistic effects on the islets, intestine, and liver^18^, ^81^ so that dorzagliatin can quickly and effectively reduce blood glucose after a meal, with less danger of hypoglycemia, and maintain good blood glucose homeostasis. It can improve the function of islets, improve the sensitivity of core glucose control organs to glucose levels, and achieve remission of glucose withdrawal.^82^

This review discusses the recent clinical progress in the use of dorzagliatin as a neo‐activating agent of GK. As an alternative therapy for T2DM, GKA are considered a potential therapy for enhancing glucose sensitivity and maintaining homeostasis in diabetic patients. The latest studies showed that dorzagliatin effectively controlled HbA1c in T2DM patients with few side effects. In addition, dorzagliatin exhibited excellent tolerability and safety and can be applied as a supplement to metformin and a single drug for the treatment of T2DM.

Dorzagliatin in combination with metformin has been used only for the treatment of patients suffering from T2DM with poor glycemic control treated with metformin alone. Dorzagliatin modulates glucose homeostasis by stimulating GK activity in the liver and pancreas and enhancing glucose‐stimulated GLP‐1 release, according to new research. When coupled with the DPP‐IV inhibitor sitagliptin, it can enhance glycemic management and cellular glucose sensitivity. Because there is no pharmacokinetic interaction between dorzagliatin and sitagliptin, dorzagliatin in combination with sitagliptin can be used to treat individuals with T2DM and obesity.^83^

However, further studies on the use of dorzagliatin in combination with other clinically important antidiabetic drugs are still lacking. Important areas for future research include the following: (1) future studies of dorzagliatin in non‐Asian populations and in a larger population of patients suffering from T2DM and (2) investigations of the possibility of dorzagliatin in combination with other conventional antidiabetic drugs, GLP‐1 receptor agonists, and SGLT2 inhibitors in patients suffering from middle and advanced T2DM, as well as the benefits and limitations of dorzagliatin in alliance with antidiabetic medicines recommended by current clinical guidelines. We believe that this development will result in more efficient management of T2DM, possibly even its remission, a decrease in the occurrence of diabetic complications, and the recovery of health and function in T2DM patients.

### CONCLUSION

Dorzagliatin can reduce blood glucose levels and insulin resistance, enhance the function of pancreatic β cells, and ameliorate hyperlipidemia and cardiovascular diseases. Dorzagliatin has demonstrated an excellent safety profile and unparalleled superiority when used alone or in combination.

## FUNDING INFORMATION

This research was funded by the Department of Science and Technology of Shandong Province; Shandong Province Key R&D Plan (grant no. 2016GSF201019), the Ji Nan science and technology Bureau; Jinan Science and Technology Innovation Program of Clinical Medicine (grant no. 201705072), the National Natural Science Foundation of China (grant no 81400788), and the Medical and Health Science and Technology Development Program of Shandong Province (grant no. 2019WS076), Postdoctoral Fund of Shandong Province (grant no. SDCX‐ZG‐202202004).

## CONFLICT OF INTEREST STATEMENT

No latent conflicts of interest were reported by the authors.
